# Grief Care Experiences of Midwives and Obstetric Nurses Following Stillbirth: A Phenomenological Study

**DOI:** 10.1002/nop2.70889

**Published:** 2026-10-09

**Authors:** Yuanling Liao, Jiaxi Xu, Aimei Mao, Xinhui Tan

**Affiliations:** ^1^ Kiang Wu Hospital Macau MAR China; ^2^ Kiang Wu Nursing College of Macau Macau MAR China

**Keywords:** grief care, midwives, obstetric nurses, phenomenology, stillbirth

## Abstract

**Aim:**

To explore midwives' and obstetric nurses' experiences in caring for families after stillbirth, understand their perspectives, identify their coping strategies, and inform improvements in grief care.

**Design:**

A qualitative phenomenological study was conducted using purposive sampling. Semi‐structured in‐depth interviews were performed with 10 midwives and obstetric nurses who had provided care to families experiencing stillbirth. Data were analysed using Colaizzi's seven‐step phenomenological analysis, with support from NVivo 12 software.

**Results:**

Midwives and obstetric nurses were profoundly affected by their experiences in caring for families who suffered stillbirths. Their experiences were categorized into three themes: Caught Up in Multiple Emotions, Silent Companionship, and Growing Through Grief‐Care. The unique features of stillbirth, combined with cultural taboos surrounding death in China, exerted a strong emotional impact on midwives and obstetric nurses. While less experienced participants tended to avoid direct communication with grieving families, senior nurses proactively engaged in silent companionship. Through reflective practice, Midwives and obstetric nurses learned to balance providing emotional support to families with personally maintaining their own emotional well‐being.

**Conclusion:**

Midwives and obstetric nurses cultivated skills and strengthened their emotional resilience through caring for families who had experienced stillbirth. Junior midwives and obstetric nurses need support in grief care for stillbirth cases.

**Implications for the Profession and/or Patient Care:**

**Impact:**

The study explored the often‐overlooked emotional experiences of nursing staff caring for families after stillbirth. It found that healthcare providers faced intense emotional distress influenced by cultural taboos, but developed coping strategies through reflection. The findings underscore the need for support programs to safeguard midwives' and nurses' well‐being and enhance grief care for bereaved families, particularly in Chinese obstetric settings.

**Reporting Method:**

The Consolidated Criteria for Reporting Qualitative Research (COREQ) checklist was used for reporting this study.

**No Patient or Public Contribution:**

No patients or members of the public were involved in the design, conduct, reporting, or dissemination of this study. The study participants were midwives and obstetric nurses.

## 1 Introduction

The definition of stillbirth varies internationally. According to the World Health Organization (2023), stillbirth is defined as the delivery of an infant without signs of life at 22 weeks of gestation or later. However, some international comparisons adopt a threshold of 28 weeks, encompassing intrauterine foetal death and death during delivery (World Health Organization 2023).

Globally, stillbirth remains a significant public health concern. World Health Organization (2023) reports approximately 1.9 million stillbirths each year, with rates ranging from 1.4 to 32.2 per 1000 live births across countries. About 84% of stillbirth cases occur in low‐ and middle‐income countries, while high‐income regions generally report rates below 2.5 per 1000 births (Hug et al. 2020). Despite declining global stillbirth rates between 2000 and the pre‐COVID‐19 pandemic period, the pandemic caused a resurgence due to disruptions in prenatal care and limited access to healthcare services (Homer et al. 2021; Watson 2020). For instance, a population‐based study in Bihar, India, documented a 24.3% increase in stillbirths during the pandemic time (Dandona et al. 2023). Grief care for families experiencing stillbirth remains a significant and evolving focus in both nursing research and clinical practice. Midwives and obstetric nurses play a critical role in providing emotional support, facilitating communication and delivering family‐centred care after stillbirth. Understanding their experiences is essential for strengthening nursing practice and improving support for bereaved families.

## 2 Background

Humanized nursing and grief counselling have advanced in fields such as oncology and palliative care, where patient death is often anticipated, and healthcare professionals can prepare for emotional challenges. Stillbirths occur suddenly and unpredictably, transforming the joyful anticipation of new life into profound grief. This traumatic transition inflicts acute emotional distress and can have long‐term consequences for psychological health and well‐being. Expectant mothers are particularly affected, and they may frequently experience shock, guilt, anxiety, anger, emotional breakdown, insomnia, decreased appetite and other emotional or physiological reactions (Endo and Saito 2024; Kurz 2020).

Midwives and obstetric nurses are often the first point of contact for families experiencing stillbirth. They have to simultaneously care for the deceased infant, support grieving families and process their own emotional responses (Boyle et al. 2020; Endo and Saito 2024). This dual burden can trigger complex emotional responses among midwives and obstetric nurses, such as grief, guilt, frustration, helplessness and anxiety (Aydın and Aktas 2021; López‐Ibernón et al. 2025; Sheehy and Baird 2022; Shen et al. 2022), yet targeted support for these professionals is limited (Jonas‐Simpson et al. 2013).

Cultural factors further shape healthcare professionals' caring experiences in stillbirth cases. In many societies, stillbirths are concealed due to stigma (Ayebare et al. 2021). In Chinese culture, public discussion of death is traditionally taboo, and mothers and families who experience stillbirth may hesitate to discuss miscarriage or stillbirth openly. This social silence can exacerbate psychological distress and prolong grieving for both bereaved families and the health professionals who care for them (Mills et al. 2021). Although existing literature has primarily focused on family needs and psychological interventions, limited attention has been paid to healthcare providers' subjective experiences and grief‐care practices (Ravaldi et al. 2022).

This gap has direct relevance to nursing practice. Midwives and obstetric nurses are expected to provide clinical care, emotional support and family‐centered grief care after stillbirth, yet limited evidence explains how they experience this role, manage their own emotions, communicate with bereaved families and identify support needs in practice. Although perinatal bereavement care guidelines emphasize sensitive communication, memory‐making, family support and staff support, less is known about how these recommendations are experienced and enacted by nurses in Chinese cultural settings where death and perinatal loss remain sensitive topics (Boyle et al. 2020; Zhuang et al. 2022). Addressing this gap can inform bereavement care training, post‐event debriefing, institutional support, and more consistent grief care for families experiencing stillbirth.

## 3 The Study

### 3.1 Aims

This study aims to explore the experiences of midwives and obstetric nurses in caring for families experiencing stillbirth. By examining their authentic experiences, this study would provide evidence‐based insights to strengthen support for healthcare professionals and optimise grief care for bereaved families.

## 4 Methods

### 4.1 Design

A phenomenological qualitative approach, which is suitable for exploring participants' lived experiences and the meanings they attach to those experiences.

### 4.2 Study Setting

This study was conducted in Macao, one of the two Special Administrative Regions of China, characterized by a distinct demographic profile and legal framework. According to the Government of Macao Special Administrative Region Statistics and Census Service (2021), ethnic Chinese account for 89.4% of the resident population. In Macao, the Penal Code (Government Printing Bureau of the Macao Special Administrative Region 1995) stipulates that selective abortion is illegal except when the pregnant woman's life or physical health is at serious risk. The law on selective abortion is strictly enforced, which contrasts with the situation in mainland China, where sex‐selective abortion remains more common despite the prohibition of non‐medical abortions.

The Macao Civil Registry defines stillbirths as the delivery of a foetus showing no signs of life at or after 22 weeks of gestation (Civil Registry Code of the Macao Special Administrative Region 2024). Despite an advanced economy and substantial government investment, stillbirths occur occasionally. The Government of Macao Special Administrative Region Statistics and Census Service (2024) recorded 7–16 stillbirth cases annually over the past five years in a population of approximately 680,000, corresponding to 1.2–3.5 per 1000 live births.

Two hospitals in Macau provide obstetric services. The study was conducted in the larger hospital and the older hospital, which has offered obstetric service for over 100 years. This hospital accounts for 59.4% of local deliveries (Government of Macao Special Administrative Region Statistics and Census Service 2024). To better support families affected by stillbirth, the hospital established a Bereavement Support Team (BST) in 2017. This team, composed of obstetric, maternal and paediatric nurses, provides comprehensive physical and psychological care to bereaved families. Members of the BST receive quarterly training sessions that include case discussions, experience sharing and workshops on evidence‐based care. In the hospital, it is recommended that BST members be prioritized to provide care for stillbirth cases. However, due to limited staffing and the unpredictable nature of stillbirths, non‐BST midwives and obstetric nurses are often involved in providing such care.

### 4.3 Participants and Recruitment

Participants were recruited through purposive sampling from the hospital's obstetrics department.

Inclusion criteria: (1) Registered midwives or obstetric nurses currently working in obstetric care. ‘Midwives’ referred to registered nursing professionals who held a midwifery qualification and were involved in labour and delivery‐related care in the delivery room; ‘Obstetric nurses’ referred to registered nurses working in the prenatal ward, postpartum ward and newborn ward (containing the intensive care unit for newborns), but did not hold a midwifery qualification; (2) those who had provided direct care to stillbirth cases. Pregnant midwives or obstetric nurses were excluded from the study to prevent potential psychological distress.

### 4.4 Data Collection

Data collection was conducted from March to September 2023 using semi‐structured in‐depth interviews. Potential participants who met the inclusion criteria were invited in person by the primary researcher, who had eight years of experience as a midwife at the hospital and was a master's degree candidate. Since admission, delivery and post‐mortem care are primarily handled by obstetric nurses, they were specifically invited to participate. All invited individuals agreed to participate. The interview guide was framed by McClelland's (1998) STAR (Situation, Task, Action, Result) model. The STAR model provides a structured framework for eliciting participants' experiences by guiding them to describe Situation (S): the context and timing of the event; Task (T): the assignment or objectives they were responsible for; Action (A): the measures they took to achieve those objectives; and Result (R): the outcomes and reflections following their actions. This model was adopted because it helps participants recall and articulate their experiences in a chronological and detailed manner, which enhances the richness and coherence of qualitative narratives. An initial interview guide was drafted, with the final version developed after rehearsal and consultation with an experienced qualitative researcher, two midwives and one nurse. The STAR‐framed interview guide is provided in Table S1. The data from participants in the pretest interviews were not included or analysed in the final results.

The primary researcher was a colleague of the participants in the same hospital, but did not hold a managerial or supervisory position over them. She acted as the interviewer and explained the study's purpose, procedures and expected time commitment. To minimize potential bias and ensure data integrity, participation was entirely voluntary, and confidentiality was emphasized during recruitment. All interviews were conducted in a non‐transparent room located outside the participants' work areas (e.g., nurses' stations or clinical offices), with no one present besides the participants and interviewer. Face‐to‐face interviews took place in a quiet room in the obstetric department, lasted 50–80 min (average 61 min), and were fully recorded. Nonverbal behaviours—such as pauses, tone changes, facial expressions and emotional reactions—were also documented, as these provided valuable contextual data for interpreting participants' experiences. No repeat interviews were conducted.

### 4.5 Data Analysis

Following each interview, the primary researcher transcribed the audio recordings into Microsoft Word within 24 h. The transcribed data were independently analysed by two researchers using Colaizzi's seven‐step method of thematic analysis, a widely recognized phenomenological framework for extracting, interpreting and validating core themes derived from participants' lived experiences (Coates 2004; see Figure 1). The coding trail was developed through a hierarchical process that began with raw data, which were assigned descriptive codes and subsequently grouped into broader themes. NVivo 12 software supported thematic coding, cross‐referencing of contextual details—including nonverbal cues—and efficient management of textual data. This facilitated traceability of coding decisions and systematic data organization and minimized potential researcher bias. Data analysis was conducted inductively, with categories and themes generated directly from participants' narratives rather than mapped onto pre‐existing STAR components. Data collection and analysis were conducted concurrently, and recruitment ended when subsequent interviews confirmed existing categories and themes without generating new meanings relevant to the research aim. This indicated sufficient meaning saturation for the present study (Hennink et al. 2017). Therefore, the sample of 10 participants was considered adequate for Colaizzi's phenomenological analysis, as this study prioritized the depth and richness of lived‐experience data rather than statistical representativeness.

**FIGURE 1:**
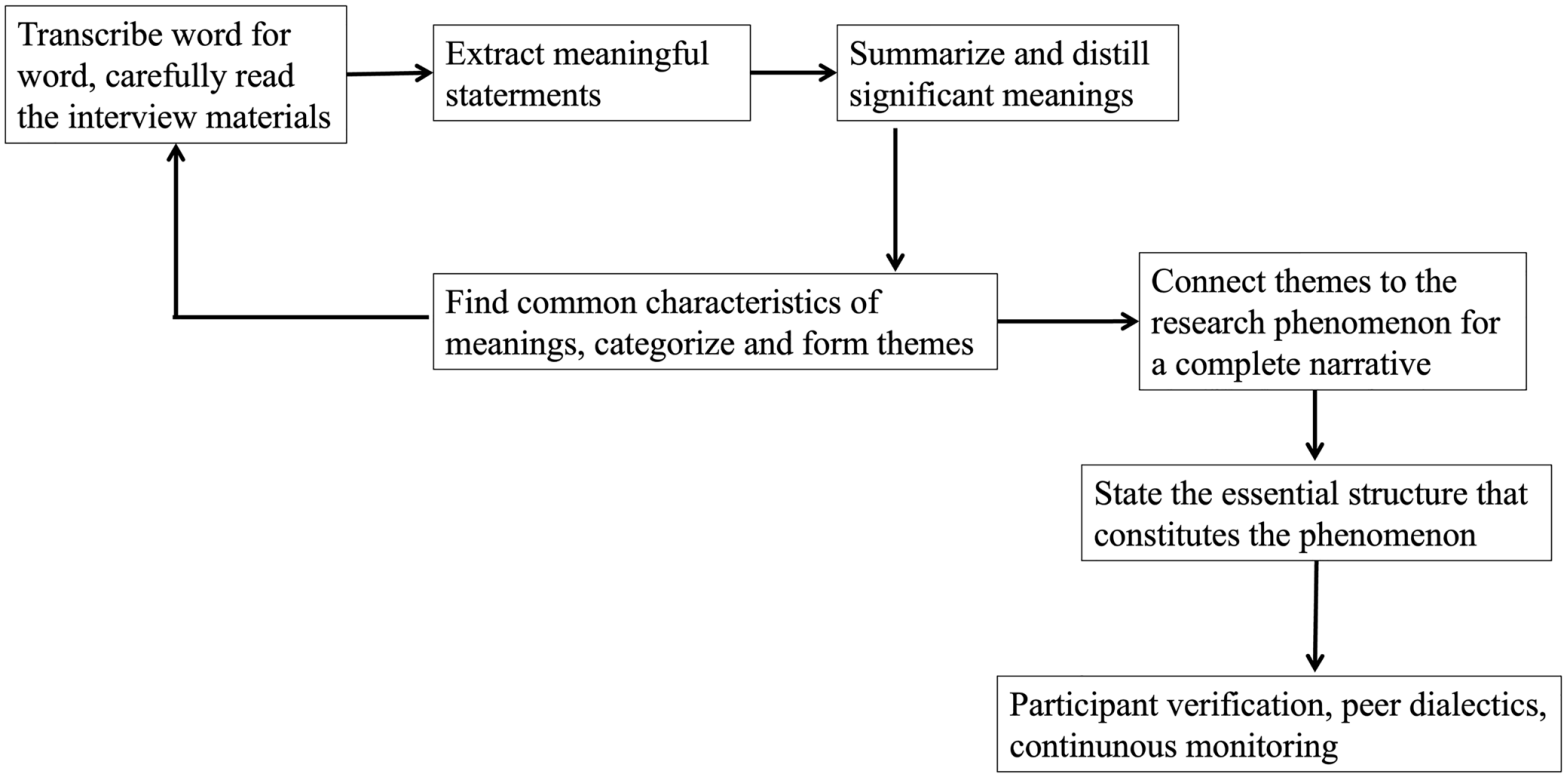
Colaizzi's seven‐step method.

### 4.6 Ethical Considerations

This study was approved by the relevant Ethics Committees (Approval No.: REC‐2022.29; KWH 2023‐005) and adhered to the ethical principles of the Declaration of Helsinki (World Medical Association 2013). Participants were fully informed about the study's purpose and procedures before the interviews were conducted. Participation was voluntary, and participants could withdraw at any time without any adverse effects imposed on them. A signed consent form from the participants was obtained before the interviews were conducted. All data, including transcripts and audio recordings, were anonymized and treated confidentially to protect participants' privacy.

### 4.7 Rigour

The study was reported in accordance with the Consolidated Criteria for Reporting Qualitative Research (COREQ) Checklist. The methodological rigour of qualitative research is commonly evaluated using four criteria—credibility, transferability, confirmability and dependability—proposed by Anfara et al. (2002). These criteria provide a robust framework for assessing qualitative study quality. In this study, the research team operationalized each criterion through specific methodological strategies, detailed in Table 1.

**TABLE 1:** Strategies employed in the study to enhance rigour.

Rigour criterion	Definition	Strategies employed
Credibility	The extent to which the study's findings accurately represent participants' lived experiences and realities.	(1) Detailed field notes documenting nonverbal cues and emotional expressions. (2) Maintenance of reflexive journals throughout data collection and analysis. (3) Member checking with three participants for transcript validation (no modifications requested). (4). Member checking with three participants for analytic verification of themes (no modifications requested).
Transferability	The degree to which the findings can be applied or transferred to other contexts with similar characteristics.	(1) Provision of rich, thick descriptions of the study context, participants, and data collection procedures. (2) Inclusion of verbatim participant quotations to illustrate analytic findings and allow readers to assess contextual applicability.
Confirmability	The extent to which findings are grounded in participants' narratives rather than researcher bias, ensured through transparency and reflexivity.	(1) Continuous peer debriefing and audit trail discussions were conducted with the supervisor, an experienced qualitative researcher and member of the research group. (2) Triangulation of data sources by including both midwives and obstetric nurses. (3) Reflexive self‐awareness of the primary researcher, a master's candidate trained in qualitative research methods, to monitor preconceptions and potential bias. (4) All members of the research team were female, which facilitated empathetic engagement and open communication with participants discussing emotionally sensitive experiences related to stillbirth, while ongoing reflexive practice ensured analytical neutrality.
Dependability	Stability and consistency of data collection, analysis, and findings over time or across researchers.	(1) Independent coding and theme generation by the primary researcher and supervisor, followed by consensus discussions. (2) Regular supervision meetings to review methodological decisions and ensure procedural consistency. (3) Maintenance of an audit trail including raw data, coded transcripts, and analytic memos.

## 5 Findings

### 5.1 Participants

Ten participants were included in the study—six midwives and four obstetric nurses—all female, aged 26–44 years. No participants withdrew from this study halfway through. Participant characteristics are presented in Table 2.

**TABLE 2:** Demographic and professional characteristics of participants (*N* = 10).

Participant ID	Age (years)	Marital status	Number of children	Professional qualification	Years of experience in obstetric care	Number of stillbirth cases involved
N1	26	Unmarried	0	Level II midwife	3–5	3–5
N2	41	Married	2	Level I midwife	> 10	> 10
N3	29	Married	1	Level II midwife	5–10	5–10
N4	44	Married	1	Level I midwife	> 10	> 10
N5	26	Unmarried	0	Level III midwife	1–3	1–3
N6	36	Married	1	Level III nurse	1–3	1–3
N7	32	Unmarried	0	Level II midwife	5–10	3–5
N8	31	Married	1	Level II nurse	5–10	3–5
N9	32	Married	2	Level II nurse	5–10	3–5
N10	32	Married	1	Level II nurse	5–10	5–10

The thematic analysis of the interviews from the 10 participants yielded 19 categories, which were ultimately clustered into three major themes. Theme 1, ‘Caught Up in Multiple Emotions’, describes the immediate emotional impact of stillbirth care on midwives and obstetric nurses. Theme 2, ‘Silent Companionship’, contains two sub‐themes—passive silence and proactive silence—which reflect different meanings and functions of silence in grief care. Theme 3, ‘Growing Through Grief‐Care’, includes two sub‐themes—‘control of environment’ and ‘control of emotions’—and illustrates how participants gradually developed more organized coping mechanisms and professional competencies (see Figure 2).

**FIGURE 2:**
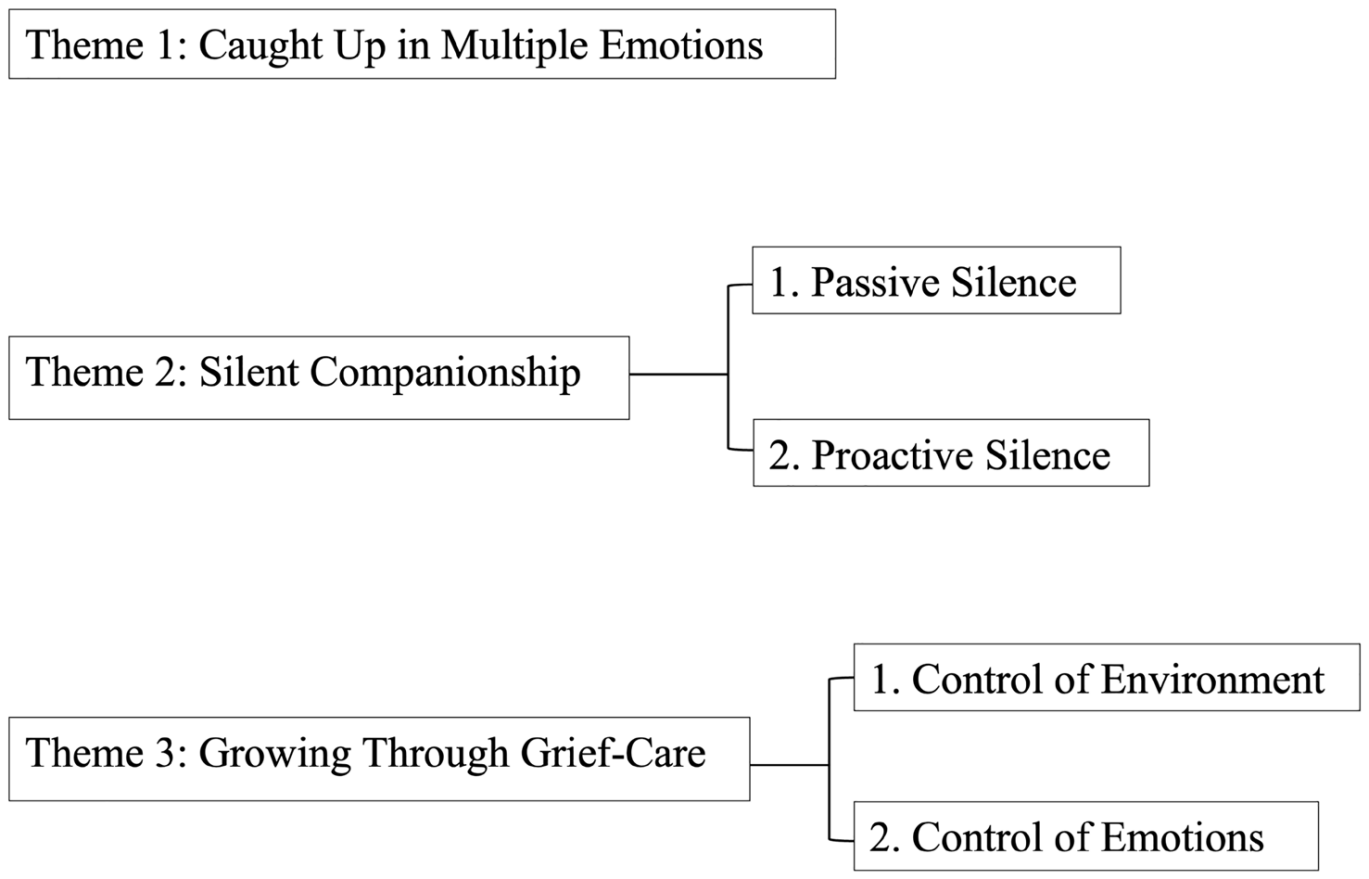
Themes and sub‐themes related to participants' experiences with caring for stillbirth cases.

### 5.2 Theme 1: Caught up in Multiple Emotions

Stillbirths, though relatively rare, are sudden and unpredictable and inflict significant psychological trauma on those midwives and obstetric nurses who are involved in caring for the cases. The midwives and obstetric nurses were impressed by almost each of the cases they encountered and could vividly recall the details. While those who encountered such cases for the first time were particularly vulnerable to intense and complex emotions, the senior who had encountered them many times felt distressed too.

N4, a midwife with over 20 years of experience in the delivery room, acknowledged that despite witnessing numerous stillbirths, each case remains emotionally challenging. She reflected, ‘I step into that role, placing myself in the mother's shoes. I stand by her side, offering constant reassurance, while inside I'm overwhelmed with grief—because after all, it's a life that's been lost’.

The abnormal appearance of a stillborn baby could have a profound visual impact on the midwives and obstetric nurses, eliciting tension and fear. N5, a midwife with three years of experience, recounted her first encounter: ‘Seeing that baby emerge from the mother's body—purple and dark, tiny, limp and slightly swollen—then wrapping it in a blanket. I am caring for the infant's body alone, with no one to assist me. I felt scared when I touched it’. N1's recollection: ‘Everyone seemed afraid and tried to avoid it (the dead foetus). They were reluctant to enter that ward unless you asked them in to help’.

Stillbirths often occur suddenly, leaving families struggling to accept the outcome and placing immense pressure on the midwives and obstetric nurses, who may worry about potential complaints or even legal actions from the families. N9, an eight‐year nurse in the prenatal ward, recounted her first experience just over a year into her career during independent night shifts. A 40‐week pregnant patient was admitted, and upon taking over, N9 detected no foetal heartbeat. She recalled, ‘I was terrified of what responsibility I might bear in the death case and had no idea what to do. Fortunately, I discovered it right after taking over. But the colleague who handed over the shift to me ended up resigning. The pressure became so overwhelming that she couldn't function normally at work’.

### 5.3 Theme 2: Silent Companionship

In case of stillbirth occurrence, the families were usually offered one bedroom despite limited ward resources. Whenever possible, members from BST were assigned to care for the families. However, non‐BST members had to stay with the family if the stillbirth happened in their shift and the BST members were not immediately available. Both BST and non‐BST members offered quiet companionship, using both passive and active forms of presence during the grief care.

#### 5.3.1 Passive Silence

Lack of training in bereavement care, combined with the infrequency of stillbirths, left some midwives and obstetric nurses unprepared to manage stillbirth situations or process their own emotions. N1, a midwife with five years of experience, recalled her feeling of loss of control the first time she encountered a stillbirth: ‘We didn't receive specialized training in this area during our internships. When you're alone with the mother who lost her baby in the delivery room, you feel at a loss—not knowing what to do or what to say to the mother’.

The participants also expressed concern about potential conflicts if grieving mothers were dissatisfied with their words or actions. They recognized that grieving families were highly sensitive to the communication provided by healthcare professionals and therefore felt the need to exercise great caution in both speech and behaviour. In such circumstances, offering quiet companionship was often perceived as a safe and appropriate form of nursing care. N10 stated, ‘I want to help, but I don't know how to help. I worry that saying the wrong thing might deepen the family's grief and cause unnecessary misunderstandings’.

#### 5.3.2 Proactive Silence

Experienced midwives and obstetric nurses, particularly the members of BST, also emphasized *silent companionship* as a crucial way of nursing care. According to their observations, families in deep sorrow were often unable to take in words of consolation. Providing a pressure‐free space allowed the families to grieve privately and to achieve psychological self‐healing. N10 (Nurse), a BST member, described her approach in which she cared for the grieving mother's choices: ‘I crouched beside her bed (since she was usually lying down), and I'd tell her, I'm here with you’. ‘*She kept crying there, so I wiped her tears away. Then I'd stroke her head or gently pat her shoulder silently*’. Her approach seemed effective, and some mothers expressed gratitude several months later for the companionship and encouragement they received from her.

### 5.4 Theme 3: Growing Through Grief‐Care

Stillbirth cases were infrequent. The midwives and obstetric nurses were impressed by each case, which turned out to be a learning opportunity for the midwives and obstetric nurses. They learned to manage their emotions, identify areas for caring improvement, optimize communication strategies and plan the best care for future cases. Ongoing training from the obstetric department further enhanced their skills, allowing them to develop personalized nursing care and provide high‐quality grief support for families.

#### 5.4.1 Control of Environment

With experience and training, midwives and obstetric nurses increasingly gained control of the environment in which they worked. They saw resources and the families as components of the environment and tried to provide a safe and private space for the grieving mothers and other family members. They learned to incorporate the holistic nursing approach with the bereaved family's background, culture, religious beliefs and social support. N3 described their lead in the holistic care: ‘We arrange a private room and allow other family members to be with the mother, avoiding placing her next to other mothers who just gave birth. Hearing healthy babies' crying can make the mother more distressed. Our (BST) team attends to specific requests—letting the mother see or dress the dead baby, writing cards, taking handprints or spending private time with the baby. We strive to fulfill these wishes’.

#### 5.4.2 Control of Emotions

Experienced midwives and obstetric nurses also learned to maintain their own emotional stability while providing support to families experiencing stillbirth. They adopted various strategies to prevent emotional exhaustion during grief care. Some nurses employed an ‘emotional distancing’ approach, consciously limiting their emotional involvement to safeguard their well‐being and sustain professional effectiveness. This is also reflected in their grief care manner in the ward. N1 (Midwife) explained, ‘We assign one or two staff members to be with the family throughout the process, aiming to minimize emotional impact. The key is to maintain personal composure—if you are overwhelmed by the grieving mother, you cannot take care of her effectively, and it affects your overall efficiency’.

On the other hand, some midwives and obstetric nurses adopted a more direct form of empathic expression—they cried alongside the grieving families. N3 (Midwife) reflected on her change from holding back tears to openly weeping with the mothers in sorrow: ‘(Nowadays) I just hold her (the mother) and we cry together. After crying, we talk—it's a form of emotional exchange. In previous times, I felt I couldn't appear unprofessional in front of patients' families, so I'd secretly go somewhere else to hold back my tears. But now I don't. I think empathy is a really good experience (light chuckle). It actually brings us closer to the mothers and their families’.

## 6 Discussion

Previous studies reported, supported by our study, that midwives and obstetric nurses often experience complex emotional responses when managing stillbirths, including sadness, guilt and worry, regardless of experience level (Altunay et al. 2025; Bayrı Bingöl et al. 2024; Endo and Saito 2024; Shen et al. 2022). Together, these findings suggest that the emotional impact of perinatal loss on healthcare professionals is not limited to a specific cultural or clinical context, but represents a broader challenge in perinatal bereavement care. Our study added that the atypical appearance of a stillborn infant provoked intense visual and emotional reactions, contributing to healthcare providers' emotional stress. Along with the appearance, other factors, such as the suddenness and unpredictability of stillbirths and cultural taboos surrounding miscarriage, also contributed to traumatic emotional responses.

Notably, our study suggested that nurse–patient conflict may exacerbate psychological burden. Nurses often face ‘self‐blame and guilt’ while fearing family accusations of negligence. Although stillbirth care provided in this study was not directly related to the specific legal restrictions on selective abortion in Macao, the broader medico‐legal and cultural context may nonetheless intensify nurses' clinical pressure. In situations where stillbirth occurs unexpectedly, and families struggle to accept the loss, nurses may fear that any inappropriate wording, delayed explanation or perceived lack of empathy might be interpreted as professional negligence. Furthermore, cultural taboos surrounding death and perinatal loss may discourage open discussion of grief, making nurses more likely to adopt cautious or avoidant communication strategies. These insights explain why some participants utilized ‘passive silence’ as a self‐protective strategy, whereas more experienced nurses successfully transformed silence into a form of compassionate presence.

Our study found profound differences between senior and junior nurses in terms of the negative emotional fluctuations. Less experienced participants were more likely to exhibit delayed responses or avoid interactions with families (Sökmen and Koç 2024), undermining the ‘patient‐centered’ care principle. Other studies have found that as years of service increase, nurses' willingness to proactively provide psychological support also rises (Salazar et al. 2022). However, our study revealed that even nurses with five years' work experience were not senior enough to develop mutual emotional management in caring for stillbirth cases. This may indicate the complexity of stillbirth‐related grief care. Heightened concerns about potential nurse–patient conflicts were found to be another motivation for passive silence. This passive coping strategy was also reported in Altunay et al.'s (2025) phenomenological study in Turkey, which showed that physicians, nurses and midwives who witnessed perinatal loss avoided caregiving or became more cautious in their practice in order to protect themselves from negative emotions. This logic of ‘inaction due to fear of error’ may temporarily avoid conflict but misses critical opportunities to provide emotional support (Begüm et al. 2021; Ssegujja et al. 2023), leaving grief care trapped in a state of inaction.

Conversely, more experienced participants in our study strategically used ‘silence’ as an active form of communication. Galla (2023) highlighted that silence can have varying effects: awkward silences may heighten discomfort, whereas compassionate silences create a reassuring environment. In our study, more experienced participants accompanied families using steady, supportive body language and attentive eye contact, providing a silent but powerful presence, in contrast to the passive silence of novice nurses driven by nervousness and uncertainty. According to Benner's (1984) ‘novice‐to‐expert’ model, novice nurses often rigidly follow rules and lack situational flexibility, relying on conservative ‘error‐avoidance’ strategies. In contrast, experienced nurses intuitively adjust care practices based on accumulated experience, transforming ‘silence’ into a compassionate, tailored approach that meets family needs (Kuo and Chen 2021). This process exemplifies the professional maturation of nurses, progressing from ‘mechanical execution’ to ‘humanistic care’.

Our study articulated two emotional coping mechanisms applied by midwives and obstetric nurses while offering grief care in stillbirth events: emotional distancing and empathic sublimation. Emotional distancing functions as a self‐protective strategy, allowing nurses to prevent emotional exhaustion while sustaining high‐quality care (Kuo and Chen 2021). Importantly, when midwives and obstetric nurses move beyond mere suppression and instead engage with families' grief through genuine empathy of crying together, this shift transforms nurses from passive emotional bearers to active emotional guides, representing a significant milestone in psychological maturity. This progression aligns with Lazarus and Folkman's (1984) stress and coping model: nurses initially experience intense stress responses, then appraise the situation and apply problem‐focused or emotion‐focused coping strategies, ultimately achieving a balanced psychological state (Kim and Choi 2020).

What is particularly valuable is that the healthcare providers in our study not only learned to regulate their own emotions but also gradually developed support skills, reflecting the phenomenon of ‘growing through grief’. This finding is consistent with Altunay et al. (2025), who described the adapted attitudes and behaviours developed by healthcare professionals to cope with negative emotions, particularly empathy toward bereaved mothers and families and the maintenance of a professional attitude during care. In our study, the midwives' and nurses' control of the environment, including assigning private rooms, avoiding shared spaces with mothers of live births, and ensuring consistent nursing staff accompaniment, helped create a low‐stimulus grief setting that prevented secondary trauma from exposure to normal newborn care. This personalized model has emerged through practical exploration, combining hands‐on experience with departmental support such as grief care training for specialized teams like the BST, gradually demonstrating measurable benefits for family psychological recovery. These findings indicate that optimizing obstetric grief care requires more than individual skill enhancement; it demands a systematic framework of departmental support, training, empowerment, and practice feedback. This process exemplifies ‘experiential learning’ in professional contexts: as theorized by Tedeschi and Calhoun (1996), individuals who endure significant trauma may experience enhanced personal strength, empathy and interpersonal skills (Henson et al. 2021).

### 6.1 Practical Implications

The findings of this study offer practical implications for obstetric nursing practice. First, grief care after stillbirth should be incorporated into routine obstetric care rather than depending only on individual nurses' experience. Consistent with existing perinatal bereavement care guidelines, hospitals should develop clear protocols to guide nurses in providing privacy, reducing bereaved mothers' exposure to routine maternity care and healthy newborns, supporting family presence and offering memory‐making opportunities, such as seeing, holding, dressing, photographing or creating mementos with the stillborn baby when culturally and clinically appropriate (Boyle et al. 2020; Smith et al. 2020; Zhuang et al. 2022).

Second, the distinction between passive silence and proactive silence observed in this study has direct implications for nursing education and institutional support. Bereavement care training should help nurses move beyond silence caused by fear or uncertainty and learn to use quiet presence, simple reassurance, attentive listening and appropriate non‐verbal support as therapeutic components of grief care. Simulation‐based training, case discussion, mentorship and post‐event debriefing may strengthen junior nurses' communication skills and emotional preparedness, particularly because previous studies have shown that health professionals often experience sadness, helplessness, fear and emotional burden when caring for families after perinatal loss (Altunay et al. 2025; Jonas‐Simpson et al. 2013; Ssegujja et al. 2023). Post‐event debriefing should also specifically address the visual impact of stillborn infants, as the atypical appearance of the baby may trigger fear, distress or intrusive memories among nurses. Such debriefing can provide a safe space for nurses to discuss these visual impressions, normalize their emotional responses and identify coping strategies for future encounters. In our study, BST members appeared to provide more structured and proactive forms of grief care in some accounts, suggesting that specialized training and repeated reflective practice may facilitate the transition from passive silence to therapeutic presence. Therefore, establishing specialized bereavement support teams while providing basic bereavement care education for all obstetric nurses may help standardize grief care, improve care consistency, and protect nurses' emotional well‐being.

### 6.2 Limitations of the Study

Given the small sample from a single hospital, findings from the study may not reflect all obstetric nurses' experiences. Participants' recollections may also be affected by recall bias.

### 6.3 Recommendations for Further Research

Grief training enhances care quality, particularly for less experienced nurses who are prone to psychological distress and reduced professional confidence following stillbirths. These findings can be used by healthcare institutions to establish robust support systems, including psychological counselling, peer support groups, post‐event debriefings and mentorship programs to guide junior staff in grief communication.

Future research should include larger and more diverse samples across regions, hospital levels and professional groups, as well as varied data collection methods to generate more comprehensive evidence. In addition, future studies could evaluate the effectiveness of visual‐trigger‐informed debriefing and simulation‐based preparation in reducing nurses' distress, strengthening emotional preparedness, and improving their confidence in stillbirth‐related grief care.

## 7 Conclusion

This study elucidates the complex psychological trajectories experienced by midwives and obstetric nurses when providing care for families experiencing stillbirth, characterized by a gradual movement from emotional turbulence to strategic adaptation. This study provides qualitative evidence for constructing a bidirectional support system between ‘caregivers and care recipients’. Findings provide qualitative evidence for establishing a bidirectional support system between caregivers and families, emphasizing the need for strengthened grief care training and institutional support. These insights enhance understanding of nurses' experiences, inform clinical practice, and underscore the importance of advancing both the professionalization and humanization of perinatal nursing.

## Author Contributions

**Yuanling Liao:** writing – original draft, writing – review and editing, conceptualization, methodology, software, data curation, formal analysis. **Jiaxi Xu:** conceptualization, writing – review and editing. **Aimei Mao:** conceptualization, methodology, software, writing – review and editing, supervision, data curation, formal analysis. **Xinhui Tan:** conceptualization, data curation.

## Funding

The authors have nothing to report.

## Disclosure

The authors confirm that any data used in the submitted manuscript were lawfully acquired in accordance with the Nagoya Protocol on Access to Genetic Resources and the Fair and Equitable Sharing of Benefits Arising from Their Utilization to the Convention on Biological Diversity. All relevant fieldwork permissions were obtained, and the corresponding permit numbers are listed.

Justification for Exceeding Reference Limit: This manuscript cites more than 25 references because it integrates both local and international literature to provide a thorough and well‐contextualized analysis of obstetric grief care. Including these sources was essential to support the discussion, substantiate findings, and ensure a comprehensive understanding of the topic.

## Ethics Statement

This study was approved by the Ethics Committees of Kiang Wu Nursing College of Macau (Approval No.: REC‐2022.29) and Kiang Wu Nursing Hospital (Approval No.: KWH 2023–005) and was conducted in accordance with the ethical principles outlined in the Declaration of Helsinki (World Medical Association 2013). Participants were fully informed about the study's purpose and procedures before the interviews were conducted. Participation was voluntary, and participants could withdraw at any time without any adverse effects imposed on them. A signed consent form from the participants was obtained before the interviews were conducted. All data, including transcripts and audio recordings, were anonymized and treated confidentially to protect participants' privacy.

## Consent

The authors have nothing to report.

## Conflicts of Interest

The authors declare no conflicts of interest.

## Supporting information


**Table S1:** The STAR‐framed interview guide.

## Data Availability

The data that support the findings of this study are available on request from the corresponding author. The data are not publicly available due to privacy or ethical restrictions.
