# Supplementary material for: Grief Care Experiences of Midwives and Obstetric Nurses Following Stillbirth: A Phenomenological Study
Source: Nurs Open. 2026 Oct 9;13(10):e70889. doi: 10.1002/nop2.70889 (PMC13652514; doi:10.1002/nop2.70889)
Supplement: Supplementary file 1 [file NOP2-13-e70889-s001.docx]

Supplementary Table S1. The STAR-framed Interview Guide

| STAR | Interview Outline |
| --- | --- |
| Situation  (S) | 1. Could you briefly describe your professional background in nursing? Including your years of experience, primary roles, and any specialized training in obstetrics.  2. How many cases of stillbirth have you encountered throughout your career? Which particular experience left the deepest impression on you, and why?  3. Can you describe the circumstances surrounding that case? How did the mother, family members, and other staff respond emotionally during that situation? |
| Task  (T) | 4. What responsibilities did you assume when caring for the mother in this case? Could you describe the step-by-step process you followed to manage the care and emotional needs of the mother and other family members? |
| Action  (A) | 5. How did your team support you in handling the situation?  6. What emotions did you experience while providing care, and how did you manage your own emotional responses? |
| Result  (R) | 7. What is your perspective on the current procedures and protocols for managing stillbirths in your ward?  8. Based on your experiences, what practical advice, strategies, or guidance would you offer to nurses or midwives encountering a stillbirth for the first time, both in terms of clinical care and emotional support?  9. Are there any significant topics, experiences, or perspectives related to stillbirth case that you feel have not been adequately explored in this interview? |
